# Solid Phase Epitaxy of Single Phase Two-Dimensional Layered InSe Grown by MBE

**DOI:** 10.3390/nano12142435

**Published:** 2022-07-15

**Authors:** Chia-Hsing Wu, Yu-Che Huang, Yen-Teng Ho, Shu-Jui Chang, Ssu-Kuan Wu, Ci-Hao Huang, Wu-Ching Chou, Chu-Shou Yang

**Affiliations:** 1 International College of Semiconductor Technology, National Yang Ming Chiao Tung University, Hsinchu 30010, Taiwan; ab708011@gmail.com (C.-H.W.); yuchehuang@nctu.edu.tw (Y.-C.H.); chia500@nctu.edu.tw (Y.-T.H.); 2 Department of Electrophysics, National Yang Ming Chiao Tung University, Hsinchu 30010, Taiwan; wusykuann@gmail.com (S.-K.W.); wcchou957@nycu.edu.tw (W.-C.C.); 3 Department of Electrical Engineering, Tatung University, Taipei 10452, Taiwan; g10702031@ms.ttu.edu.tw

**Keywords:** InSe, solid-phase epitaxy, MBE, In_2_Se_3_

## Abstract

Single-phase two-dimensional (2D) indium monoselenide (γ-InSe) film is successfully grown via solid phase epitaxy in the molecular beam epitaxy (MBE) system. Having high electron mobility and high photoresponsivity, ultrathin 2D γ-InSe semiconductors are attractive for future field-effect transistor and optoelectronic devices. However, growing single-phase γ-InSe film is a challenge due to the polymorphic nature of indium selenide (γ-InSe, α-In_2_Se_3_, β-In_2_Se_3_, γ-In_2_Se_3_, etc.). In this work, the 2D α-In_2_Se_3_ film was first grown on a sapphire substrate by MBE. Then, the high In/Se ratio sources were deposited on the α-In_2_Se_3_ surface, and an γ-InSe crystal emerged via solid-phase epitaxy. After 50 min of deposition, the initially 2D α-In_2_Se_3_ phase was also transformed into a 2D γ-InSe crystal. The phase transition from 2D α-In_2_Se_3_ to γ-InSe was confirmed by Raman, XRD, and TEM analysis. The structural ordering of 2D γ-InSe film was characterized by synchrotron-based grazing-incidence wide-angle X-ray scattering (GIWAXS).

## 1. Introduction

Recently, two-dimensional (2D) transition metal dichalcogenides (TMDs) have attracted strong interest from the scientific community because of their unique electrical and optical properties, making them promising candidates for future electronic and optoelectronic applications [1,2]. In addition to 2D TMDs, 2D III-VI metal chalcogenides such as In_2_Se_3_ and InSe were also shown potential applications in electronic devices [3,4,5] and optoelectronic devices [6,7,8]. Particularly, InSe with a six monolayer device exhibiting ultra-high mobility of 1000 cm^2^V^−1^s^−1^ at room temperature reported by Denis A. Bandurin et al. [9] shows a promising candidate to surpass silicon technology in the future. Although the exfoliation methods have been widely used to investigate 2D III-VI semiconductors’ exceptional electrical properties, synthesizing large-area high-quality layered materials still requires bottom-up strategies, which are more suitable to be realized for industrial applications. To date, epitaxial growth by chemical vapor deposition (CVD), pulsed laser deposition (PLD), or molecular beam epitaxy (MBE) has been used for the synthesis of 2D III-VI metal chalcogenides [10,11,12,13,14,15]. However, the synthesis of single-phase indium selenide is still a challenge by chemical vapor deposition (CVD). Because indium selenide has multiple structures (γ-InSe, α-In_2_Se_3_, β-In_2_Se_3_, γ-In_2_Se_3_, etc.) that co-exist at room temperature [16,17,18]. To be implemented for industrial applications, the growth of indium selenide film with a single-phase conducted by precise control of In and Se sources is required. Among the techniques used to fabricate 2D semiconductors, MBE is widely considered one of the most competitive, precisely controlling the ultra-low growth rate and making the grown film clean for device fabrication [19,20,21].

In this study, the MBE system successfully demonstrated the epitaxial 2D γ-InSe thin film via solid-phase epitaxy. The Raman, X-ray diffraction (XRD), and high-resolution transmission electron microscopy (HR-TEM) image show that the 2D α-In_2_Se_3_ fully converted to 2D γ-InSe. We believe that the 2D γ-InSe growth mechanism is solid-phase epitaxy due to similar in-plane lattice and space groups between 2D α-In_2_Se_3_ and 2D γ-InSe.

## 2. Materials and Methods

**Figure 1:**
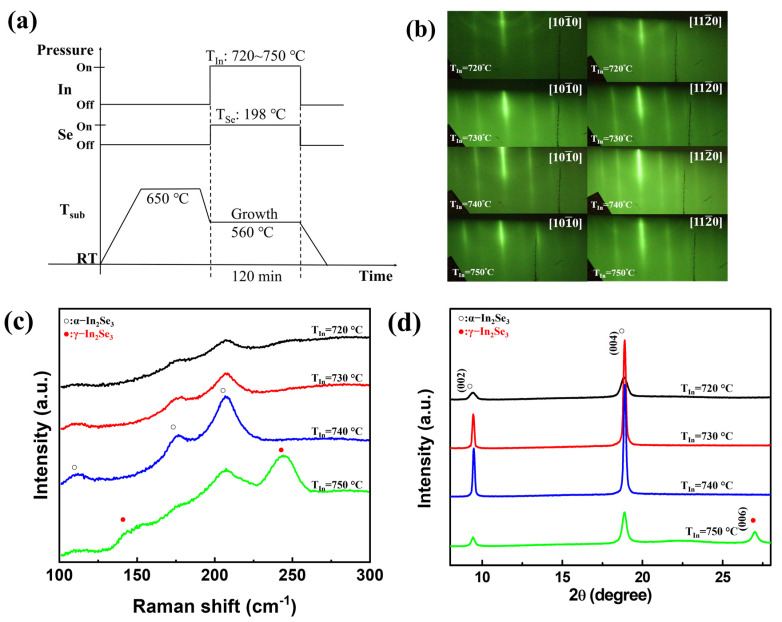
(**a**) The process flow of In_2_Se_3_ growth. (**b**) The RHE ED patterns of In_2_Se_3_ along with [10$\bar{1}$0] (on the **left**) and [11$\bar{2}$ 0] (on the **right**) arimuth of *T_In_* varied at 720 °C, 730 °C, 740 °C, and 750 °C, respectively. (**c**) Raman spectra and (**d**) XRD θ-2θ scans of In_2_Se_3_ films with various *T_In_* temperatures.

Indium selenide films were deposited on c-plane sapphire substrates using a homemade MBE system in an ultra-high vacuum chamber with a base pressure of 2 × 10^−8^ Torr. In and Se sources were evaporated using Knudsen cells (K-cells), and the growth processes were monitored using in-situ RHEED(R-DEC Co., Ltd., Ibaraki, Japan). Before the deposition, the sapphire substrates were degreased in acetone by ultrasonic cleaning for 2 min, and then a mixture of phosphoric acid and sulfuric acid of 1:3 for 15 min. Finally, the substrates were immersed in deionized water for 2 min. After the chemical cleaning process, the substrates were introduced to the growth chamber, and the sapphire substrate was thermal cleaned at 650 °C for 30 min. To identify the growth conditions for α-In_2_Se_3_ growth, the K-cells temperature of In (*T_In_*) was varied at 720 °C, 730 °C, 740 °C, and 750 °C, and Se (*T_Se_*) was fixed at 198 °C, denoted In/Se ratio 0.05, 0.06, 0.07, and 0.11, respectively. Detailed calculation of In/Se ratio has been shown in Appendix A. The growth time of all samples was 2 h and the growth process schematic was shown in Figure 1a. The optimal α-In_2_Se_3_ film was achieved with In (*T_In_*) at 740 °C and substrate temperature (*T_sub_*) of 560 °C, resulting in growth rates of 0.58 nm/min. After having single-phase α-In_2_Se_3_ film, the In and Se sources were deposited on the α-In_2_Se_3_ surface by closing the Se shutter as a high In/Se ratio at *T_In_* of 740 °C, *T_Se_* of 198 °C, *T_sub_* of 560 °C The crystallographic properties of the α-In_2_Se_3_ and γ-InSe were examined using XRD (D8, Bruker corp. Billerica, MA, USA) system and synchrotron-based grazing-incidence wide-angle X-ray scattering (GIWAXS) at Taiwan Photon Source (TPS) TPS25A (National Synchrotron Radiation Research Center, Hsinchu, Taiwan). The GIWAXS data were collected with an area detector. High-resolution transmission electron microscopy (HR-TEM) (ARM200F, JEOL Ltd., Tokyo, Japan) was used to probe the α-In_2_Se_3_ and γ-InSe microstructure and obtain atomic-scale images. The Raman spectra were obtained using a 532 nm wavelength of solid-state laser (70 mW) as the excitation source and a triple grating spectrometer (iHR-550, HORIBA, Ltd., Kyoto, Japan) system as a signal detector.

## 3. Results and Discussion

Figure 1b presents the in-situ RHEED patterns of In_2_Se_3_ with *T_In_* varied from 720 °C to 750 °C, and *T_Se_* fixed at 198 °C. As seen in the RHEED pattern, the stripe patterns were obtained along with [10$\bar{1}$0] and [11$\bar{2}$0] azimuth, indicating that In_2_Se_3_ was in layer-by-layer growth mode. When the *T_In_* was lower than 740 °C, both the ring and streaky lines were exhibited in the RHEED pattern, indicating the layered and polycrystalline structures of In_2_Se_3_ were formed. Raman spectroscopy and XRD were used to identify the various indium selenide structures [22,23,24,25,26,27]. Figure 1c shows the Raman spectra of In_2_Se_3_ thin film on the sapphire substrate grown by *T_In_* varied from 720 °C to 750 °C. The vibration modes of In_2_Se_3_ at *T_In_* at 740 °C exhibit three peak features at 112, 176, and 207 cm^−1^, which were characteristic of α-In_2_Se_3_ [11,12,22,23,24,25]. When the *T_In_* was up to 750 °C, the vibrational mode appeared with two additional peaks at 146 cm^−1^ and 244 cm^−1^, which were characteristic of γ-In_2_Se_3_ [11,12,22,23]. Figure 1d shows the XRD θ-2θ scans of In_2_Se_3_ grown by *T_In_* varied from 720 °C to 750 °C. The (002) and (004) α-In_2_Se_3_ diffraction peaks are at 9.43° and 18.89°, and the peak of (006) γ-In_2_Se_3_ is at 27.03°. As *T_In_* at 740 °C, the optimal α-In_2_Se_3_ films were achieved because of the relatively high diffraction intensity and narrower full width at half maximum (FWHM)of 0.16° of the α-In_2_Se_3_ peaks. The crystal grain size is estimated as 52.6 nm from the Scherrer equation. However, the *T_In_* at 750 °C leads to the co-existence of both α-In_2_Se_3_ and γ-In_2_Se_3_ structures, which confirmed our Raman analysis that the film grown by *T_In_* set at 750 °C had both α-In_2_Se_3_ and γ-In_2_Se_3_ vibration modes.

**Figure 2:**
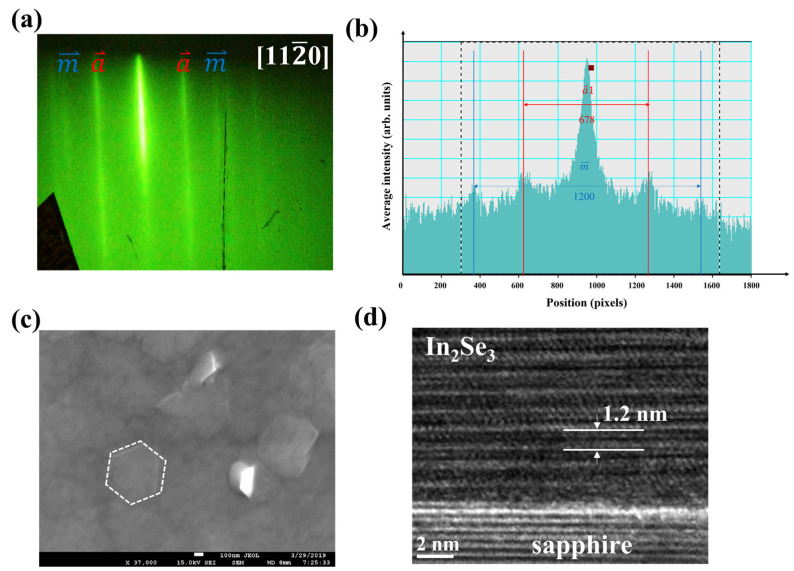
(**a**) The RHEED pattern of In_2_Se_3_ films grown by T_In_ at 740 °C, where $\;\vec{a}$ and $\vec{m}$ are indexed as diffraction from [10$\bar{1}$0] and [11$\bar{2}$0]. (**b**) intensity profile of (**a**). (**c**) A top-view SEM image of the α-In_2_Se_3_ film. (**d**) A cross-sectional HR-TEM image of the α-In_2_Se_3_ film.

To gain more insight into the α-In_2_Se_3_ crystal configurations and microstructure, one may examine the streaky line spacing in the RHEED pattern. Figure 2a,b shows the RHEED pattern and intensity profile of optimal α-In_2_Se_3_ films, and $\vec{a}$ and $\vec{m}$ are (10$\bar{1}$0) and (11$\bar{2}$0). The calculated $\vec{a}/\vec{m}$ is close to $\sqrt{3}$, where the $\vec{a}-\vec{a}$ spacing is 678 pixels and $\vec{m\;}$−$\vec{\;m}$ spacing is 1200 pixels, revealing that the α-In_2_Se_3_ crystalline layer is six-fold symmetry on the sapphire substrate. Figure 2c,d shows the SEM image and cross-sectional HR-TEM image of the α-In_2_Se_3_ microstructure. The shape of the α-In_2_Se_3_ grains exhibited a hexagonal pattern, and the microstructure of α-In_2_Se_3_ exhibited a layered structure on the sapphire, where the α-In_2_Se_3_ monolayer thickness is 1.2 nm. The growth mechanism of the α-In_2_Se_3_ on the sapphire substrate may be similar to quasi-van der Waals (vdW) epitaxy [28,29,30].

**Figure 3:**
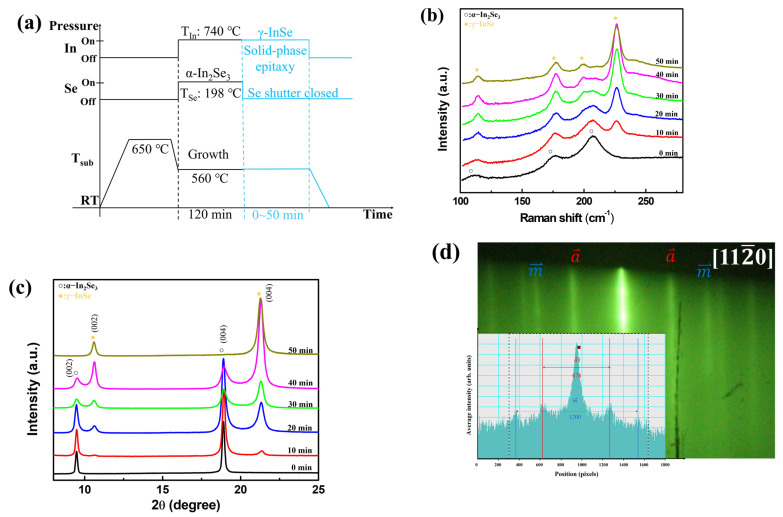
(**a**) The process flow of γ-InSe growth via solid-phase epitaxy. (**b**) The Raman spectra and (**c**) XRD θ-2θ scans of γ-InSe films with the high In/Se ratio at various deposition times. (**d**) The RHEED pattern of γ-InSe with a high In/Se ratio at 50 min deposition time. The inset of the figure shows the intensity profile of the RHEED.

**Figure 4:**
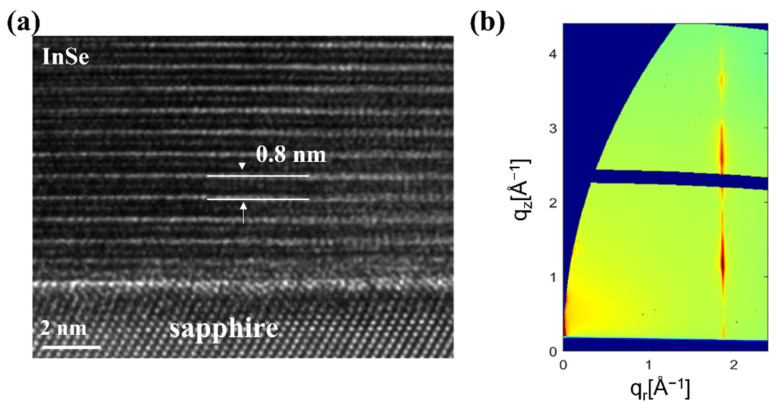
(**a**) Cross-sectional HR-TEM image and (**b**) 2D GIWAXS profile of γ-InSe films with high In/Se ratio at 50 min deposition time.

**Figure 5:**
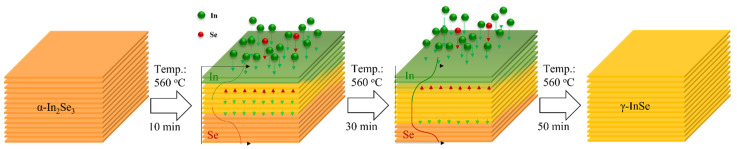
Schematic illustration showing growth mechanism of single-phase γ-InSe film via solid-phase epitaxy.

From the above observations, we found that the single-phase α-In_2_Se_3_ could rely on simply controlling In/Se ratio, and we noted that the in-plane lattice of γ-InSe (4.01 Å) is close to that of α-In_2_Se_3_ (4.03 Å) and has the same space groups. Therefore, we proposed the solid-phase epitaxy method to grow single-phase γ-InSe films, which uses a single crystalline α-In_2_Se_3_ film as a template, followed by depositing In and Se sources for γ-InSe crystal growth on the α-In_2_Se_3_ and then heating the film to crystallize it. Figure 3a shows the process flow of InSe growth via solid-phase epitaxy. The optimal *α*-In_2_Se_3_ films were achieved with *T_In_* set at 740 °C and a growth temperature of 560 °C. Then, the *T_In_* and T_Se_ were kept, and the K-cell of the Se shutter was closed to become a high In/Se ratio with deposition times of 10 min to 50 min. Figure 3b presents the Raman spectra of InSe growth with high In/Se ratios for various deposition times. The vibration modes of γ-InSe appeared on the α-In_2_Se_3_ surface after the high In/Se ratio was deposited for 10 min, but when the deposition time reached 50 min, only the γ-InSe vibration signal remained. Figure 3c shows the XRD θ-2θ scans of InSe growth with high In/Se ratios for 10 min to 50 min deposition times. The (002) and (004) γ-InSe diffraction peaks emerged while the deposition time exceeded 10 min, and the peak intensity of γ-InSe gradually increased with the deposition time. In contrast, the peak intensity of α-In_2_Se_3_ gradually decreased with the deposition time. When the deposition time reached 50 min, the sample had only γ-InSe peaks. The FWHM of (004) γ-InSe peaks is around 0.36°, and the grain size is estimated as 23.5 nm. The Raman and XRD results show that solid-phase epitaxy on the α-In_2_Se_3_ surface effectively grows single-phase γ-InSe. Interestingly, *α*-In_2_Se_3_ also phase transitions to γ-InSe, probably because of the high diffusivity of Indium. Figure 3d presents the RHEED pattern of γ-InSe with high In/Se ratios for 50 min deposition time. The inset of Figure 3d shows the intensity profile of γ-InSe. In addition to showing the stripe pattern of γ-InSe in the RHEED pattern, the $\vec{a}$−$\;\vec{a}$ and $\vec{m}$−$\vec{m}$ spacing of γ-InSe are 672 pixels and 1150 pixels, which is similar to that of α-In_2_Se_3_ in Figure 2b. This confirmed the similar lattice spacing of γ-InSe and α-In_2_Se_3_. Figure 4a shows the cross-sectional HR-TEM image of γ-InSe with high In/Se ratios for 50 min deposition time. The microstructure of γ-InSe exhibited a layered structure and well-defined crystal lattice, in which the monolayer thickness of 0.8 nm is only two-thirds of α-In_2_Se_3_. Even the *α*-In_2_Se_3_, initially on the sapphire substrate, was transformed into the γ-InSe phase. To verify these local microstructure observations, we utilized GIWAXS measurement to investigate the structure of low-dimension materials and provide information on the structural morphology of an entire sample [31,32]. Figure 4b presents the 2D reciprocal space map of the γ-InSe films via the solid-phase epitaxy. The lateral face of (10$\bar{1}$0) γ-InSe is at q_r_ = 1.88 Å^−1^. Since the γ-InSe is layered in nature and the crystal dimension is confined along the out-of-plane direction, the γ-InSe shows a vertical stripe pattern along the crystal truncation rod (CTR) in reciprocal space. Figure 5 illustrates the growth of γ-InSe via solid-phase epitaxy as a model. The single-phase α-In_2_Se_3_ film grown via MBE system at 560 °C, followed by the high In/Se ratio deposition on the α-In_2_Se_3_ film, the films underwent a thermodynamic transformation into γ-InSe crystal growth on the *α*-In_2_Se_3_ surface. The formation energy for InSe and In_2_Se_3_ are −0.527 and −0.322 eV/atom [33,34]. The practical indium selenide growth requires kinetic considerations such as growth temperature and concentration of sources [22]. During the γ-InSe crystal growth as a function of deposition times, the transformation mechanism involved the α-In_2_Se_3_ to the γ-InSe transition, and the whole structure finally became a single-phase γ-InSe structure.

## 4. Conclusions

This work demonstrated the single-phase γ-InSe epitaxy via solid-phase epitaxy in an MBE system. The α-In_2_Se_3_ was first grown on a sapphire substrate by MBE as a template, and then the γ-InSe was formed by depositing In and Se at a high In/Se ratio on the α-In_2_Se_3_ films. The growth mechanism of γ-InSe was facilitated by a close in-plane lattice match between the α-In_2_Se_3_ and γ-InSe. Raman and XRD measurement revealed that γ-InSe emerged on the α-In_2_Se_3_ structure, and the α-In_2_Se_3_ phase transformed into γ-InSe during the high In/Se ratio deposition. Furthermore, HR-TEM and GIWAXS measurements showed that the whole structure finally becomes the single-phase 2D γ-InSe structure. The solid-phase epitaxy method opens the door to the development of 2D materials and the possibility of making more 2D materials available for electronic device applications.

## Figures and Tables

**Figure A1:**
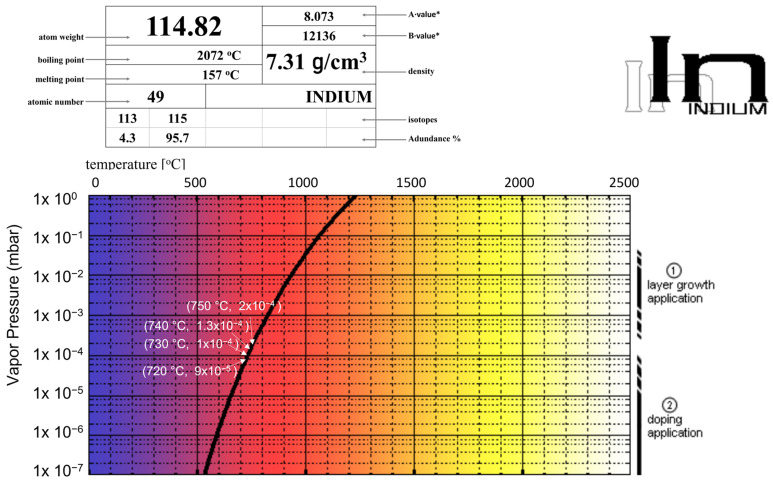
The temperature dependent vapor pressure of In.

**Figure A2:**
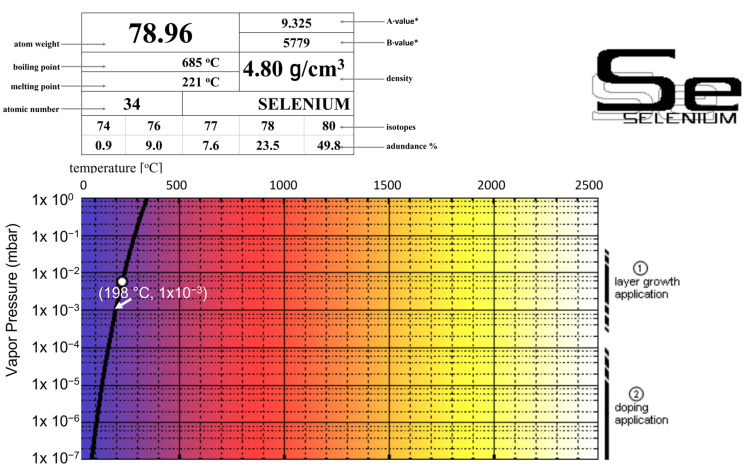
The temperature dependent vapor pressure of Se.

## Data Availability

The data presented in this study are available on request from the corresponding authors.

## Appendix A

The In/Se ratio is estimated from the vapor pressure and cell temperature. The vapor pressure of In and Se are referenced from Eberl MBE-Komponenten GmbH [35,36], as shown in Figure A1 and Figure A2. The evaporation rate calculation of material is referenced from Andrew Sarangan [37]. The evaporation rate of the material can be written as:

$$Z_{A}=3.5\times 10^{22}\frac{\;P}{\sqrt{M\times T}}$$

where

*Z_A_* is the rate in atoms/cm^2^/s

*P* is the vapor pressure in Torr

*T* is the vapor temperature in Kelvin

*M* is the molar mass

The calculated evaporation rate (Z_Se(In)_) and In/Se ratio are shown in the table below. The *T_In_* was varied at 720 °C, 730 °C, 740 °C, and 750 °C, and *T_Se_* was fixed at 198 °C; that In/Se growth ratio was varied from 0.05, 0.06, 0.07, and 0.011.

**Table A1:** The In/Se ratio calculation referenced from the formula of evaporation rate of the materials [37].

Material	Mole Mass M	Temperature (°C)	Vapor Pressure (mbar)	Temperature (K)	Za = *p*/(M*T(K))^0.5^	Ratio = Za(In)/Za(Se)
Se	79	198	1.00 × 10^−3^	471	5.18413 × 10^−6^	
In	115	720	9.00 × 10^−5^	993	2.66329 × 10^−7^	0.051373951
In	115	730	1.00 × 10^−4^	1003	2.94443 × 10^−7^	0.056796897
In	115	740	1.30 × 10^−4^	1013	3.80881 × 10^−7^	0.073470621
In	115	750	2.00 × 10^−4^	1023	5.831 × 10^−7^	0.112477915

## References

[1] Wang Q.H., Kalantar-Zadeh K., Kis A., Coleman J.N., Strano M.S. (2012). Electronics and Optoelectronics of Two-Dimensional Transition Metal Dichalcogenides. Nat. Nanotechnol..

[2] Mak K.F., Shan J. (2016). Photonics and optoelectronics of 2D semiconductor transition metal dichalcogenides. Nat. Photonics.

[3] Sánchez-Royo J.F., Muñoz-Matutano G., Brotons-Gisbert M., Martínez-Pastor J.P., Segura A., Cantarero A., Mata R., Canet-Ferrer J., Tobias G., Canadell E. (2014). Electronic structure, optical properties, and lattice dynamics in atomically thin indium selenide flakes. Nano Res..

[4] Feng W., Zheng W., Cao W., Hu P. (2014). Back Gated Multilayer InSe Transistors with Enhanced Carrier Mobilities via the Suppression of Carrier Scattering from a Dielectric Interface. Adv. Mater..

[5] Sucharitakul S., Goble N.J., Kumar U.R., Sankar R., Bogorad Z.A., Chou F., Chen Y., Gao X.P.A. (2015). Intrinsic Electron Mobility Exceeding 10³ cm²/(V s) in Multilayer InSe FETs. Nano Lett..

[6] Tamalampudi S.R., Lu Y.-Y., U R.K., Sankar R., Liao C.-D., Boopathi K.M., Cheng C.-H., Chou F.C., Chen Y.-T. (2014). High Performance and Bendable Few-Layered InSe Photodetectors with Broad Spectral Response. Nano Lett..

[7] Lei S., Ge L., Najmaei S., George A., Kappera R., Lou J., Chhowalla M., Yamaguchi H., Gupta G., Vajtai R. (2014). Evolution of the electronic band structure and efficient photo-detection in atomic layers of InSe. ACS Nano.

[8] Lei S., Wen F., Li B., Wang Q., Huang Y., Gong Y., He Y., Dong P., Bellah J., George A. (2015). Optoelectronic Memory Using Two-Dimensional Materials. Nano Lett..

[9] Cantrell D.A. (2017). High electron mobility, quantum Hall effect and anomalous optical response in atomically thin InSe. Nat. Nanotechnol..

[10] Zhang X., Lee S., Bansal A., Zhang F., Terrones M., Jackson T.N., Redwing J.M. (2020). Epitaxial growth of few-layer β-In2Se3 thin films by metalorganic chemical vapor deposition. J. Cryst. Growth.

[11] Küpers M., Konze P.M., Meledin A., Mayer J., Englert U., Wuttig M., Dronskowski R. (2018). Controlled Crystal Growth of Indium Selenide, In2Se3, and the Crystal Structures of α-In_2_Se_3_. Inorg. Chem..

[12] Huang W., Gan L., Li H., Ma Y., Zhai T. (2018). Phase-Engineered Growth of Ultrathin InSe Flakes by Chemical Vapor Deposition for High-Efficiency Second Harmonic Generation. Chem. Eur. J..

[13] Zheng D., Shiogai J., Fujiwara K., Tsukazaki A. (2018). Pulsed-laser deposition of InSe thin films for the detection of thickness-dependent bandgap modification. Appl. Phys. Lett..

[14] Yang Z., Jie W., Mak C., Lin S., Lin H., Yang X., Yan F., Lau S.P., Hao J. (2017). Wafer-Scale Synthesis of High-Quality Semiconducting Two-Dimensional Layered InSe with Broadband Photoresponse. ACS Nano.

[15] Hsiao S., Yang C., Yang H., Wu C., Wu S., Chang L., Ho Y., Chang S., Chou W. (2022). Novel Method for the Growth of Two-Dimensional Layered InSe Thin Films on Amorphous Substrate by Molecular Beam Epitaxy. Front. Mater..

[16] Rashid R., Ling F.C.-C., Wang S.-P., Xiao K., Cui X., Ki Q.R.D.-K. (2021). IP and OOP ferroelectricity in hexagonal γ-In_2_Se_3_ nanoflakes grown by chemical vapor deposition. J. Alloys Compd..

[17] Hu Y., Feng W., Dai1 M., Yang H., Chen X., Liu G., Zhang S., Hu P. (2018). Temperature-dependent growth of few layer β-InSe and α-In2Se3 single crystals for optoelectronic device. Semicond. Sci. Technol..

[18] Balakrishnan N., Steer E.D., Smith E.F., Kudrynskyi Z.R., Kovalyuk Z.D., Eaves L., Patanè A., Beton P.H. (2018). Epitaxial growth of γ-InSe and α, β, and γ-In_2_Se_3_ on ε-GaSe. 2D Mater..

[19] Vishwanath S., Liu X., Rouvimov S., Basile L., Lu N., Azcatl A., Magno K., Wallace R.M., Kim M., Idrobo J.-C. (2016). Controllable growth of layered selenide and telluride heterostructures and superlattices using molecular beam epitaxy. J. Mater. Res..

[20] Seredyński B., Ogorzałek Z., Zajkowska W., Bożek R., Tokarczyk M., Suffczyński J., Kret S., Sadowski J., Gryglas-Borysiewicz M., Pacuski W. (2021). Molecular Beam Epitaxy of a 2D Material Nearly Lattice Matched to a 3D Substrate: NiTe_2_ on GaAs. Cryst. Growth Des..

[21] Pacuski W., Grzeszczyk M., Nogajewski K., Bogucki A., Oreszczuk K., Kucharek J., Połczyńska K.E., Seredyński B., Rodek A., Bożek R. (2020). Narrow Excitonic Lines and Large-Scale Homogeneity of Transition-Metal Dichalcogenide Monolayers Grown by Molecular Beam Epitaxy on Hexagonal Boron Nitride. Nano Lett..

[22] Han G., Chen Z., Drennan J., Zou J. (2014). Indium Selenides: Structural Characteristics, Synthesis and Their Thermoelectric Performances. Small.

[23] Segura A. (2018). Layered Indium Selenide under High Pressure: A Review. Crystals.

[24] Grimaldi I., Gerace T., Pipita M.M., Perrotta I.D., Ciuchi F., Berger H., Papagno M., Castriota M., Pacilé D. (2020). Structural investigation of InSe layered semiconductors. Solid State Commun..

[25] Waghmare A., Sharma V., Shinde P., Punde A., Vairale P., Hase Y., Pandharkar S., Nair S., Aher R., Doiphode V. (2022). Preparation and characterization of γ-In2Se3 thin-film photoanodes for photoelectrochemical water splitting. J. Solid State Electrochem..

[26] Lewandowska R., Bacewicz R., Filipowicz J., Paszkowicz W. (2001). Raman scattering in α-In2Se3 crystals. Mater. Res. Bull..

[27] Sun Y., Pang S., Zhang J. (2022). Layer Number-Dependent Raman Spectra of γ-InSe. J. Phys. Chem. Lett..

[28] Liang D., Wei T., Wang J., Li J. (2020). Quasi van der Waals epitaxy nitride materials and devices on two dimension materials. Nano Energy.

[29] Liu C., Huang H., Choi W., Kim J., Jung K., Sun W., Tansu N., Zhou W., Kuo H., Li X. (2020). Hybrid Integration of n-MoS2/p-GaN Diodes by Quasi-van der Waals Epitaxy. ACS Appl. Electron. Mater..

[30] Mortelmans W., Gendt S., Heyns M., Merckling C. (2021). Epitaxy of 2D chalcogenides: Aspects and consequences of weak van der Waals coupling. Appl. Mater. Today.

[31] Chang S., Wang S., Huang Y., Chih J.H., Lai Y., Tsai Y., Lin J., Chien C., Tang Y., Hu C. (2022). van der Waals epitaxy of 2D h-AlN on TMDs by atomic layer deposition at 250 °C. Appl. Phys. Lett..

[32] Franz D., Blanc N., Coraux J., Renaud G., Runte S., Gerber T., Busse C., Michely T., Feibelman P.J., Hejral U. (2016). Atomic Structure of Pt nanoclusters supported by graphene/Ir(111) and reversible transformation under CO exposure. Phys. Rev. B.

[33] The Open Quantum Materials Database (OQMD). https://oqmd.org/materials/composition/InSe.

[34] The Open Quantum Materials Database (OQMD). https://oqmd.org/materials/composition/In2Se3.

[35] Eberl MBE-Komponenten GmbH. https://www.mbe-komponenten.de/selection-guide/element/in.php.

[36] Eberl MBE-Komponenten GmbH. https://www.mbe-komponenten.de/selection-guide/element/se.php.

[37] Sarangan A. (2016). Physical and Chemical Vapor Deposition. Nanofabrication, Principles to Laboratory Practice.

